# From curved spacetime to spacetime-dependent local unitaries over the honeycomb and triangular Quantum Walks

**DOI:** 10.1038/s41598-019-47535-4

**Published:** 2019-07-29

**Authors:** Pablo Arrighi, Giuseppe Di Molfetta, Ivan Marquez-Martin, Armando Perez

**Affiliations:** 1 Aix-Marseille Univ, Université de Toulon, CNRS, LIS, Marseille, France and IXXI, Lyon, France; 2 Aix-Marseille Univ, Université de Toulon, CNRS, LIS, Marseille, France and Departamento de Física Teórica and IFIC, Universidad de Valencia-CSIC, Dr. Moliner 50, 46100 Burjassot, Spain; 3 Departamento de Física Teórica and IFIC, Universidad de Valencia-CSIC, Dr. Moliner 50, 46100 Burjassot, Spain

**Keywords:** Theoretical physics, Quantum simulation

## Abstract

A discrete-time Quantum Walk (QW) is an operator driving the evolution of a single particle on the lattice, through local unitaries. In a previous paper, we showed that QWs over the honeycomb and triangular lattices can be used to simulate the Dirac equation. We apply a spacetime coordinate transformation upon the lattice of this QW, and show that it is equivalent to introducing spacetime-dependent local unitaries —whilst keeping the lattice fixed. By exploiting this duality between changes in geometry, and changes in local unitaries, we show that the spacetime-dependent QW simulates the Dirac equation in (2 + 1)–dimensional curved spacetime. Interestingly, the duality crucially relies on the non linear-independence of the three preferred directions of the honeycomb and triangular lattices: The same construction would fail for the square lattice. At the practical level, this result opens the possibility to simulate field theories on curved manifolds, via the quantum walk on different kinds of lattices.

## Introduction

### Quantum walks

QWs are quantum dynamical systems characterized by: *(i)* a state space which is restricted to the one-particle sector (i.e. to a single ‘walker’); *(ii)* a discrete spacetime; *(iii)* the unitarity of its evolution; *(iv)* the homogeneity of its evolution, meaning its translation-invariance and time-independence, and *(v)* its causality (i.e. it is ‘non-signalling’), meaning that information has a bounded speed of propagation. QWs are blossoming, for two good reasons.

The first is that a number of novel Quantum Computation algorithms, to be run on Quantum Computers, were discovered via QWs^1,2^, or were elegantly expressed using QWs (the Grover search for instance). Typically in these quantum algorithms, the QW explores a graph, whose shape encodes the instance of the problem. No continuous spacetime limit is taken in these works.

The second is that a number of novel Quantum Simulation schemes, to be run on quantum simulation devices, were first expressed as QWs^3,4^, which seems to be the natural language for doing so. Quantum simulation was Feynman’s initial motivation to invent Quantum Computing^5^. Whilst full-blown Quantum Computers remain out-of-reach at the experimental level, a number of special-purpose Quantum Simulation devices are appearing, whose architecture is often directly inspired by QWs^6,7^. In QW-based quantum simulation schemes, the quantum walker propagates on a grid, and a spacetime continuum limit towards some well-known target physics equation is taken. These schemes provide: a/ numerical schemes that are stable even for classical computers— from which one can derive convergence^8^; b/simple toy models of the target physical phenomena, with most symmetries conserved (homogeneity, causality, unitarity… sometimes even Lorentz-covariance^9,10^.

The present work falls within the second class. However it borrows from the first. Indeed, we describe depart from the square lattice, to go to the honeycomb and triangular lattice— which can be seen as trivalent graphs.

### Rationale

A motivation for this work is the possibility to describe and implement the quantum simulation of certain physical systems, without the need to rely on the square lattice architecture. Rather, one would like to phrase a quantum simulation scheme in terms of naturally occurring lattices in well-controlled substrates. Examples of this class are the simulation of condensed matter systems modeled by a tight-binding Hamiltonian, such as graphene^11^ or the Kagome lattices^12^— where the dynamics of electrons can be effectively recast as a Dirac-like equation. In fact the QW introduced in this paper may be useful as a simple point of departure to predict electronic transport properties in the graphene like-materials^13^ and exploring how varying their geometry may influence the dispersion relations, and lead to topological phases^14^, with interesting consequences on the conducting properties.

Another motivation for this work is to understand how fermions would propagate if spacetime were a triangulated manifold, at the fundamental level. Indeed, triangulated manifolds are being used to describe curved spacetime since^15^– when Regge introduced his simplicial, discrete formulation of General Relativity. This discrete formulation then motivated a number of quantum gravity theories, such as Loop Quantum Gravity^16^ and Causal Dynamical Triangulation^17^— which seek to recover Regge calculus in the classical limit. Most often quantum gravity research focuses on the core issue of the quantum dynamics of discrete spacetime itself— overlooking the question of how matter would propagate within the discrete spacetime structure it prescribes. The present ideas may help address the question.

### Duality

In a previous work, we showed how a QW can be defined on the honeycomb and the triangular lattice^18^ (see also^19^), whose continuous spacetime limit is the Dirac equation in (2 + 1)– dimensional spacetime. Here, we extend these definitions to allow for spacetime dependent local unitaries, and introduce a dynamics that, in the continuum limit, corresponds to the Dirac equation in a curved (2 + 1)– dimensional spacetime.

The construction, we feel, is interesting. Indeed, given a lattice made of equilateral triangles, we begin by distorting the metric just via a coordinate transformation, following the initial step of the derivation of the Dirac equation in ordinary curved spacetime. But then we realize that the coordinate transformation can be absorbed by a suitable choice of the three gamma matrices that are associated to the three directions provided by the triangles— a possibility offered by the fact that these three directions are, of course, linearly-dependent in the plane. Recall that the role of the gamma matrices is to prescribe a basis of the spin, in which spin up goes one way, and spin down goes the opposite way. In the QW, the local unitaries implement precisely the corresponding changes of base. Thus, the gamma matrices determine the local unitaries in the QW. This, therefore, unravels an equivalence, in the continuum limit, between changing the actual geometry of the lattice, or keeping it fixed but changing the local unitaries in a suitable manner. The final step is to allow the local unitaries to be spacetime dependent and take the continuum limit, thereby recovering the Dirac equation in curved spacetime.

Notice that having three directions in two-dimensional space, as in the honeycomb or triangular lattices, is what provides that extra degree of freedom allowing for the transfer of the geometric distortions into the local unitaries— the square lattice is too rigid in this respect.

### Related works

It is already well known that QW can simulate the Dirac equation^3,4,8,20–23^, the Klein-Gordon equation^24–26^ and the Schrödinger equation^27,28^ and that they are a minimal setting in which to simulate particles in some inhomogeneous background field^29–33^, with the difficult topic of interactions initiated in^34,35^. Eventually, the systematic study of the impact inhomogeneous local unitaries also gave rise to QW models of particles propagating in curved spacetime. This line of research was initiated by a QW simulations of the curved Dirac equation in (1 + 1)–dimensions, for synchronous coordinates^30,36^, and later extended by^37^ to any spacetime metrics, and generalized to further spatial and spin dimensions in^38,39^. A related work, from a slightly different perspective, can be found in^40^. All of these models were on the square lattice: to the best our knowledge no one had modeled fermionic transport over non-square lattices. The present paper shows that over the honeycomb and triangular lattices the problem becomes considerably simpler, and the solution elegant.

In a recent work^41^, quantum transport over curved spacetime has been compared to electronic transport in deformed graphene, where a pseudo-magnetic field emulates an effective curvature in the tight-binding Hamiltonian (see also^42^). Back to the quantum computing side, the Grover search has been expressed as a QW over the honeycomb lattice^43^ (see also^44^ for continuous time approach). Reference^45^ evaluates the use graphene nanoribbons as a substrate to build quantum gates.

### Plan

The paper is organized as follows. First, we remind the reader of the basic concepts and notations surrounding the Dirac equation in a curved spacetime, in (3 + 1) and (2 + 1)– dimensions. In Methods we revisit our earlier Dirac QW on a honeycomb and on a triangular lattice, and why it worked. Also we show how a simple, homogeneous coordinate transformation impacts the continuum limit of the Dirac QW. In the end of this section, it is shown the duality, i.e. how the coordinate transformation can be absorbed into a choice of local unitaries. Finally, we present the main results: a QW that reproduces the Dirac equation with curvature in the continuum limit, both for the honeycomb and for the triangular lattices. We use $\hslash =c=1$ units.

## Dirac Equation in Curved Spacetime: a Recap

In this Section we recall the basic properties of the Dirac equation in curved spacetime. We refer the reader to^46–48^ for a review. We start by describing the case of a (3 + 1)– dimensional spacetime with coordinates *x*^*μ*^, *μ* = 0, …4, where *x*^0^ is the time coordinate, and metric tensor *g*_*μν*_(*x*) in these coordinates. At each point *x*, it is possible to introduce a set of four vectors $\{{{e}_{\mu }}^{a}(x)/a,\mu =0,\,\ldots 4\}$, referred to as the tetrad or vierbein, that locally diagonalizes the metric tensor i.e.,

1 $${g}_{\mu \nu }(x)={{e}_{\mu }}^{a}(x){{e}_{\nu }}^{b}(x){\eta }_{ab}.$$

(here and thereafter, summation over repeated indices is assumed), where *η*_*ab*_ = Diag(1, −1, −1, −1). Notice that, given a vierbein, one can obtain a new one, which would also satisfy Eq. (1), by performing an arbitrary Lorentz transformation. The inverse of the vierbein is denoted ${{e}^{\mu }}_{a}$ (interchanged indices), satisfying

2 $${{e}^{\mu }}_{a}(x){{e}_{\nu }}^{a}(x)={\delta }_{\nu }^{\mu },\,{{e}_{\mu }}^{a}(x){{e}^{\mu }}_{b}(x)={\delta }_{b}^{a}.$$

Using (1) and (2), one has

3 $${g}_{\mu \nu }(x){{e}^{\mu }}_{a}(x)\,{{e}^{\nu }}_{b}(x)={\eta }_{ab}.$$

Thus, tetrads can be understood as normalized tangent vectors that relate the original coordinates to a local inertial frame. We use the common convention that inertial coordinates are designated by latin indices, and original coordinates by greek indices. Latin indices are lowered and raised by *η*_*ab*_, greek indices by *g*_*μν*_. In the local inertial frame, one is legitimated to use the Dirac *γ*– matrices, i.e. matrices satisfying the Clifford algebra $\{{\gamma }^{a},{\gamma }^{b}\}=2{\eta }^{ab}{\mathbb{I}}$. From these, one defines ${\sigma }^{ab}=\frac{i}{2}[{\gamma }^{a},{\gamma }^{b}]$.

Given a Dirac field *ψ*(*x*), the action of a local Lorentz transformation ${{{\rm{\Lambda }}}^{a}}_{b}(x)$ can be written as

4 $$\psi \to {U}_{{\rm{\Lambda }}}\psi ,$$

where

5 $${U}_{{\rm{\Lambda }}}(x)={e}^{-\frac{i}{4}{\theta }_{ab}(x){\sigma }^{ab}},$$

and *θ*_*ab*_(*x*) are the parameters of the transformation, defined by ${{{\rm{\Lambda }}}^{a}}_{b}(x)={\delta }_{b}^{a}+{{\theta }^{a}}_{b}(x)$. One can prove that this operator acts on Dirac gamma matrices as follows:

6 $${{U}_{{\rm{\Lambda }}}}^{-1}{\gamma }^{a}{U}_{{\rm{\Lambda }}}={{{\rm{\Lambda }}}^{a}}_{b}{\gamma }^{b}.$$

With the above notations, the Dirac equation in curved space

7 $$i{\gamma }^{a}{{e}^{\mu }}_{a}(x){{\mathscr{D}}}_{\mu }\,\psi -m\,\psi =0,$$

where *m* is the particle mass, is invariant under a local Lorentz transformation provided the generalized derivative that we use is

8 $${{\mathscr{D}}}_{\mu }={\partial }_{\mu }+{{\rm{\Gamma }}}_{\mu },$$

where Γ_*μ*_ transforms according to

9 $${{\rm{\Gamma }}}_{\nu }\to {{\rm{\Gamma }}}_{\nu }^{^{\prime} }={U}_{{\rm{\Lambda }}}{{\rm{\Gamma }}}_{\nu }{U}_{{\rm{\Lambda }}}^{-1}-{\partial }_{\nu }({U}_{{\rm{\Lambda }}}){U}_{{\rm{\Lambda }}}^{-1}.$$

The correction Γ_*μ*_ to the derivative can then be obtained as^47^

10 $${{\rm{\Gamma }}}_{\mu }(x)=-\,\frac{i}{4}{\omega }_{ab\mu }(x){\sigma }^{ab},$$

where *ω*_*abμ*_(*x*) is the so-called spin connection, and can be expressed in terms of the tetrads and the affine connection as

11 $${{\omega }^{a}}_{b\nu }={{e}_{\mu }}^{a}{\partial }_{\nu }{{e}^{\mu }}_{b}+{{e}_{\mu }}^{a}{{e}^{\sigma }}_{b}{{\rm{\Gamma }}}_{\sigma \nu }^{\mu }.$$

From Eq. (7) one can define a four-vector current

12 $${j}^{\mu }=\sqrt{g}{{e}^{\mu }}_{a}\bar{\psi }{\gamma }^{a}\psi ,$$

where *g* is the (absolute value of) the determinant of the metric, so that it is conserved:

13 $${\partial }_{\mu }{j}^{\mu }=0.$$

This justifies the normalization condition

14 $$\int {j}^{0}dv=\int \sqrt{g}{{e}^{0}}_{0}{\psi }^{\dagger }\psi dv=1,$$

with *dv* the volume element in space.

(2 + 1)*– dimensions*. When the space dimension is lower than 3, the *γ*–matrices become 2 × 2. Then, the Dirac Eq. (7) can be simplified to give

15 $$i{{\gamma }}^{a}[{{e}^{\mu }}_{a}{\partial }_{\mu }\psi +\frac{1}{2\sqrt{g}}{\partial }_{\mu }({{e}^{\mu }}_{a}\sqrt{g})\psi ]-m\psi =0.$$

We will now express this equation in Hamiltonian form. We name the greek indices *μ* = *t*, *x*, *y*, and the latin indices *a* = 0, 1, 2. By performing a local Lorentz transformation, it is possible to arrive to a form of the tetrad such that ${{e}^{t}}_{a}=0$ for *a* = 1, 2. Then, by introducing the change of wavefunction given by^49^:

16 $$\chi ={g}^{1/4}{({{e}^{t}}_{0})}^{1/2}\psi $$

and multiplying Eq. (15) by *β* ≡ *γ*^0^, one gets

17 $$i{\partial }_{t}\chi +\frac{i}{2}\{{B}^{s},{\partial }_{s}\}\chi -\frac{m}{{{e}^{t}}_{0}}\beta \chi =0,$$

where *s* = 1, 2, and we have introduced the notation ${B}^{s}={\alpha }^{a}\frac{{{e}^{s}}_{a}}{{{e}^{t}}_{0}}$, with the usual Dirac *α*–matrices *α*^*a*^ ≡ *βγ*^*a*^. In particular, one can make the choice *γ*^0^ = *σ*^*z*^, *γ*^1^ = *iσ*^*y*^ and *γ*^2^ = −*iσ*^*x*^. Then *α*^0^ becomes the identity matrix, *α*^1^ = *σ*^*x*^ and *α*^2^ = *σ*^*y*^, with *σ*^*i*^ (*i* = 1, 2, 3) the Pauli matrices.

According to Eqs (14) and (16), the normalization condition becomes simply

18 $$\int {\chi }^{\dagger }\chi dv=1.$$

## Methods

### Dirac QW

A possible representation of the Dirac equation in flat spacetime is obtained from Eq. (17) by using the canonical tetrads ${{e}^{\mu }}_{a}={\delta }_{a}^{\mu }$ and the choice of Dirac *α*–matrices made at the end of the last section:

19 $$i{\partial }_{t}\psi (t)={H}_{D}\psi (t)\,{\rm{with}}\,{H}_{D}={p}_{x}{\sigma }^{x}+{p}_{y}{\sigma }^{y}+m{\sigma }^{z}.$$

where *p*_*i*_ is the *i*^*th*^ component of the momentum operator.

It is now very well-known that one can define a QW on the lattice that converges, in the limit of both the lattice spacing and the time step going to zero, towards the solutions of (19). This is done by defining a Hilbert space ${\mathcal H} ={ {\mathcal H} }_{x}\otimes { {\mathcal H} }_{y}\otimes { {\mathcal H} }_{c}$, where ${ {\mathcal H} }_{x}\otimes { {\mathcal H} }_{y}$ stands for the space degrees of freedom, as spanned by the basis states *x* = *εj*, *y* = *εk* with $j,k\in {\mathbb{Z}}$, whereas ${ {\mathcal H} }_{c}={\rm{Span}}\{|c\rangle /c\in \{-1,1\}\}$ describes the internal ‘coin’ (spin) degree of freedom. Over ${ {\mathcal H} }_{x}\otimes { {\mathcal H} }_{y}$, the *p*_*i*_ will now denote the quasimomentum operators defined by

20 $$\begin{array}{rcl}\exp (-i\varepsilon {p}_{x})|x,y\rangle  & = & |x+\varepsilon ,y\rangle \\ \exp (-i\varepsilon {p}_{y})|x,y\rangle  & = & |x,y+\varepsilon \rangle .\end{array}$$

The Dirac QW will evolve a state *ψ*(*t*) into

21 $$\begin{array}{rcl}\psi (t+\varepsilon ) & = & \exp (-im\varepsilon {\sigma }^{z})\exp (-i\varepsilon {p}_{x}{\sigma }^{x})\exp (-i\varepsilon {p}_{y}{\sigma }^{y})\\  & \approx  & \exp (-i\varepsilon {H}_{D})\psi (t)\end{array}$$

using the Trotter-Kato formula. It follows that one recovers the Dirac Eq. (19) in the continuum limit when *ε* goes to zero, where the *p*_*i*_ become the true momentum operators *p*_*i*_ = −*i*∂_*i*_.

Recently^18^ we showed that Dirac dynamics can be implemented by a QW, not only over square lattices, but also over the honeycomb and triangular lattices (see also^19^). The honeycomb lattice QW is easier to introduce. It defines three directions *u*_*i*_, *i* = 0, 1, 2 having relative angles of 120°, let ${u}_{i}^{j}$ denote their coordinates. The idea is to introduce three unitary 2 × 2–matrices *τ*^*i*^ with eigenvalues ±1 such that *H*_*D*_ can be written as

22 $${H}_{D}={\pi }_{i}{\tau }^{i}+m{\sigma }^{z},$$

where ${\pi }_{i}\equiv {u}_{i}^{j}{p}_{j}$ represents the quasimomentum operator along the *u*_*i*_ direction. Then, the corresponding QW can again be defined by a Lie-Trotter expansion of Eq. (21), with *H*_*D*_ defined in (22). The triangular lattice QW makes use of a similar setup, although the translations are generated by rotations of the triangles themselves, bringing apart the internal components of the field *ψ*, which is assumed to ‘live’ in the edges of the triangles, one component (*ψ*^↑^ or *ψ*^↓^) on each side.

### Coordinate transformation on the dirac equation

The construction of the Dirac equation in curved spacetime relies on the equivalence principle, which means that one can introduce a local transformation of coordinates at a given point, so that one recovers the flat equation in the neighborhood of that point. The curved Dirac equation is then that which stems from applying reverse the local transformation, upon the flat Dirac equation. Our line of thought follows that step, i.e., starting from the flat case Dirac QW, perform an arbitrary change of coordinates so as to obtain the curved Dirac QW. Let us begin with just an homogeneous change of coordinates on the Dirac equation.

First notice that Eq. (3) can be writen as *e*^*T*^*ge* = *η*, where *e* and *g* are just the representation of the tetrads and metric in matricial form, and ^*T*^ denotes the matrix transpose. Now, under a global change of coordinates Γ such that *x*′ = Γ*x*, the metric *g* and the vierbein transform as

23 $$\begin{array}{rcl}g\,\mapsto g^{\prime}  & = & {({{\rm{\Gamma }}}^{T})}^{-1}g{{\rm{\Gamma }}}^{-1}\\ e\,\mapsto e^{\prime}  & = & {\rm{\Gamma }}e\end{array}$$

This transformation fulfills the tetrads-metric relation,

24 $${e}^{^{\prime} T}g^{\prime} e^{\prime} ={e}^{T}{{\rm{\Gamma }}}^{T}{({{\rm{\Gamma }}}^{T})}^{-1}g{{\rm{\Gamma }}}^{-1}{\rm{\Gamma }}e={e}^{T}ge=\eta .$$

Next we start from a QW that reproduces the flat equation, and introduce a deformation (described by the transformation Γ) that will end up with a more generic metric *g*′. We can make a simple choice, given by the canonical tetrads ${{e}^{\mu }}_{a}={\delta }_{a}^{\mu }$ for the initial coordinates, and then transform them according to Eq. (23). Since we are considering a deformation of the spatial sites of the lattice, the time components will be left unchanged, and the matrix Γ will take the form

25 $${\rm{\Gamma }}=(\begin{array}{lll}1 & 0 & 0\\ 0 & {\lambda }_{11} & {\lambda }_{12}\\ 0 & {\lambda }_{21} & {\lambda }_{22}\end{array}).$$

where each *λ*_*ij*_ are position independent, although they are allowed to depend on time.

Under this restriction, we can reduce the problem to a transformation on a bidimensional space, where ${{e}^{t}}_{0}=1$, which implies that Eq. (17) adopts the simpler form

26 $$i{\partial }_{t}\chi +\frac{i}{2}\{{B}^{s},{\partial }_{s}\}\chi -m\beta \chi =0.$$

Let us consider how this transformation will affect the QW defined on a triangular lattice, as introduced in Sect. III (see^18^). Such transformation will imply modifying the vectors *u*_*i*_, yielding the new vectors

27 $${u}_{i}^{^{\prime} }=(\begin{array}{ll}{\lambda }_{11} & {\lambda }_{12}\\ {\lambda }_{21} & {\lambda }_{22}\end{array}){u}_{i}\equiv {\rm{\Lambda }}{u}_{i}.$$

Introducing these vectors in our algorithms and calculating the continuum limit, we arrive at the following equation

28 $$i{\partial }_{t}\psi =[({\lambda }_{11}{\sigma }^{x}+{\lambda }_{12}{\sigma }^{y}){p}_{x}+({\lambda }_{21}{\sigma }^{x}+{\lambda }_{22}{\sigma }^{y}){p}_{y}]\psi +m{\sigma }^{z}\psi ,$$

which describes the Dirac equation on a flat geometry. A comparison with Eq. (17) gives

29 $${B}^{x}={\lambda }_{11}{\sigma }^{x}+{\lambda }_{12}{\sigma }^{y}$$

30 $${B}^{y}={\lambda }_{21}{\sigma }^{x}+{\lambda }_{22}{\sigma }^{y}.$$

This procedure can be used for a homogeneous transformation, such as the one defined above. In the next section, we introduce an alternative, which consists in redefining the *τ*^*i*^ matrices. As we shall see, this redefinition also allows for an inhomogeneous (i.e., space-time dependent) Λ(*t*, *x*, *y*) transformation, thereby resulting in a Dirac equation in curved space.

### Curved dirac equation from a non-homogeneous QW

We now generalize the ideas developed in the previous Sect. with the purpose to obtain, in the continuum limit, the Dirac equation on a curved spacetime, for a given metrics with a triangular tetrad, as discussed in Sect. II. We start by looking at the set of matrices ${B}^{s}={\alpha }^{a}\frac{{{e}^{s}}_{a}}{{{e}^{t}}_{0}}$, as a linear transformation over the set of usual Pauli matrices, in the same spirit as Eqs (29) and (30). This leads us to define the transformation Λ(*t*, *x*, *y*), with matrix elements

31 $${{\rm{\Lambda }}}_{a}^{s}\equiv \frac{{{e}^{s}}_{a}}{{{e}^{t}}_{0}}$$

(we have omitted the time and space dependence for convenience). Then, the above mentioned transformation reads

32 $${B}^{s}={{\rm{\Lambda }}}_{a}^{s}{\alpha }^{a}.$$

We now make use of the property that relates the *τ*^*i*^ matrices, defined in Eq. (22), with the Pauli matrices: ${u}_{i}^{k}{\tau }^{i}={\sigma }^{k}$ (see^18^). In this way, we arrive to

33 $${B}^{s}={{\rm{\Lambda }}}_{k}^{s}{u}_{i}^{k}{\tau }^{i}.$$

The above equation can be understood as a transformation performed on the *u*_*i*_ vectors, c.f. Eq. (27), as the origin of the curved spacetime equation.

Instead of introducing a distortion Λ(*t*, *x*, *y*) on the lattice via the modification of the *u*_*i*_ vectors, the unitary matrices *τ*^*i*^ can be transformed to produce the same effect. In other words, we seek for a set of matrices *β*^*i*^(*t*, *x*, *y*) that fulfill the following conditions:

- (C1) We impose that
  34 $${{\rm{\Lambda }}}_{k}^{j}(t,x,y){u}_{i}^{k}{\tau }^{i}={u}_{i}^{j}{\beta }^{i}(t,x,y).$$
- (C2) Each of them has {−1, 1} as eigenvalues, i.e. at any time step and at any point (*x*, *y*) of the lattice there exist three unitaries *U*_*i*_(*t*, *x*, *y*) such that

35 $${\beta }^{i}(t,x,y)={U}_{i}^{\dagger }(t,x,y){\sigma }^{z}{U}_{i}(t,x,y).$$

Notice that condition (C1) implies that the coordinate transformation dictated by ${{\rm{\Lambda }}}_{k}^{j}(t,x,y)$ is transferred to the unitary operations, which become new spacetime dependent *β*^*i*^(*t*, *x*, *y*), instead of the original *τ*^*i*^. Additionally, condition (C2) will allow us to rewrite the QW evolution in terms of the usual state-dependent translation operators. Let us apply these ideas to the honeycomb and the triangular lattice.

To alleviate the notations, in what follows we will omit the spacetime dependence both in these matrices and in the *U*_*i*_(*t*, *x*, *y*), and write simply *β*^*i*^ and *U*_*i*_. The above conditions allow to calculate the *β*^*i*^ matrices, which can be written as a combination of Pauli matrices, i.e. ${\beta }^{i}={\overrightarrow{n}}^{i}\cdot \overrightarrow{\sigma }$, where each ${\overrightarrow{n}}^{i}$ must be a real, unit vector ${\overrightarrow{n}}^{i}=(\sin \,{\theta }_{i}\,\cos \,{\varphi }_{i},\,\sin \,{\theta }_{i}\,\sin \,{\varphi }_{i},\,\cos \,{\theta }_{i})$ for some angles *θ*_*i*_ and *ϕ*_*i*_ (that are time and position dependent).

In this way

36 $${\beta }_{i}={U}_{i}^{\dagger }{\sigma }_{z}{U}_{i}=(\begin{array}{cc}\cos \,{\theta }_{i} & {e}^{-i{\varphi }_{i}}\,\sin \,{\theta }_{i}\\ {e}^{i{\varphi }_{i}}\,\sin \,{\theta }_{i} & -\cos \,{\theta }_{i}\end{array}),$$

and each *U*_*i*_ can be obtained by diagonalization of the corresponding *β*^*i*^. With an appropriate choice of phases, we finally write them as

37 $${U}_{i}=(\begin{array}{cc}{e}^{\frac{i{\varphi }_{i}}{2}}\,\cos \,\frac{{\theta }_{i}}{2} & {e}^{-\frac{i{\varphi }_{i}}{2}}\,\sin \,\frac{{\theta }_{i}}{2}\\ -{e}^{\frac{i{\varphi }_{i}}{2}}\,\sin \,\frac{{\theta }_{i}}{2} & {e}^{-\frac{i{\varphi }_{i}}{2}}\,\cos \,\frac{{\theta }_{i}}{2},\end{array}).$$

Before we proceed to examine the induced QW on the honeycomb and triangular lattices together with their limits, let us discuss what the situation would have been in the square lattice, had we implement the above procedure. In this case, the original Dirac matrices can be chosen to be the Pauli matrices, and the two unit vectors *u*_*i*_ can be taken to be the canonical ones, so that the requirement of Eq. (34) simply becomes

38 $${{\rm{\Lambda }}}_{k}^{j}{\sigma }^{k}={\beta }^{j}.$$

But then, since condition (C2) implies that det(*β*^*j*^) = −1 for each *j*, we need that

39 $$\sum _{k}\,{({{\rm{\Lambda }}}_{k}^{j})}^{2}=1.$$

Thus the square lattice only allows for a limited form of “duality”, i.e. only those transformations satisfying condition (39) can be absorbed into the unitaries, whereas the honeycomb and triangular lattices allow for arbitrary transformations.

## Results

### Honeycomb QW

In this section we define the QW over the honeycomb, following a similar procedure as in^18^. After the ideas developed in Methods, we define the following Hamiltonian to be used in the QW:

40 $$ {\mathcal H} =\frac{1}{2}{u}_{i}^{j}({\beta }^{i}{p}_{j}+{p}_{j}{\beta }^{i})+\tilde{m}{\sigma }^{z}$$

with $\tilde{m}=m/{{e}^{t}}_{0}$. Expanding the Hamiltonian, we arrive to:

41 $$ {\mathcal H} =-\,i{u}_{i}^{j}{U}_{i}^{\dagger }{\sigma }_{z}{\partial }_{j}{U}_{i}-\frac{i}{2}{u}_{i}^{j}[({\partial }_{j}{U}_{i}^{\dagger }){\sigma }_{z}{U}_{i}-{U}_{i}^{\dagger }{\sigma }_{z}({\partial }_{j}{U}_{i})]+\tilde{m}{\sigma }^{z}$$

After substitution of Eq. (37), one obtains

42 $$({\partial }_{j}{U}_{i}^{\dagger }){\sigma }_{z}{U}_{i}-{U}_{i}^{\dagger }{\sigma }_{z}({\partial }_{j}{U}_{i})=-\,i\,\cos \,{\theta }_{i}{\partial }_{j}{\varphi }_{j}{\mathbb{I}},$$

with ${\mathbb{I}}$ the identity matrix. Notice that, unlike in the flat space situation, there is no possible choice of the phases in the *U*_*i*_s that makes Eq. (42) vanish for all values of *i*. One may wonder whether there is a reason behind this, for example the existence of some topological or gauge invariant that forbids all these quantities to be simultaneously zero. This issue might deserve further investigation in the future. In any case, the additional term in Eq. (42) that arises from the choice given by Eq. (37) contributes only as a space-time dependent phase, which is easy to handle both from the theoretical and from the experimental point of view. We finally arrive to:

43 $$ {\mathcal H} =\sum _{i}\,({U}_{i}^{\dagger }{\sigma }_{z}{\pi }_{i}{U}_{i}+{\gamma }_{i}{\mathbb{I}})+\tilde{m}{\sigma }^{z}$$

where ${\gamma }_{i}=-\,\frac{i}{2}\,\cos \,{\theta }_{i}{\pi }_{i}{\varphi }_{i}$. In order to define the QW, we make use of the Lie-Trotter product formula to decompose the evolution of the wavefunction $\psi (t+\varepsilon )={e}^{-i\varepsilon  {\mathcal H} }\psi (t)$ as a product of unitary matrices

44 $${e}^{-i\varepsilon [\sum _{i}({U}_{i}^{\dagger }{\sigma }_{z}{\pi }_{i}{U}_{i}+{\gamma }_{i})+\tilde{m}{\sigma }^{z}]}\approx {e}^{-i\tilde{m}\varepsilon {\sigma }^{z}}\prod _{i}\,{e}^{-i\varepsilon {U}_{i}^{\dagger }{\sigma }_{z}{\pi }_{i}{U}_{i}}{e}^{-i\varepsilon {\gamma }_{i}}.$$

Applying condition (C1), and introducing the translation operators along the *u*_*i*_ direction as ${T}_{i}={e}^{-i\varepsilon {\sigma }^{z}{\pi }_{i}}$, the QW on a honeycomb can be defined as:

45 $$\psi (t+\varepsilon )={e}^{-i\tilde{m}\varepsilon {\sigma }^{z}}\prod _{i}\,{{U}^{\dagger }}_{i}{T}_{i}{U}_{i}{e}^{-i\varepsilon {\gamma }_{i}}\psi (t)$$

By construction, in the continuous limit, we arrive to the Dirac equation in 2 + 1 curved space-time, under the form

46 $$i{\partial }_{t}\psi =\frac{1}{2}[{u}_{i}^{j}{\beta }^{i}(t,x,y){p}_{j}+{u}_{i}^{j}{p}_{j}{\beta }^{i}(t,x,y)]\psi +\tilde{m}{\sigma }^{z}\psi .$$

As expected, this equation can be nicely rewritten under the form Eq. (17), if we define ${B}^{j}(t,x,y)\equiv {u}_{i}^{j}{\beta }^{i}(t,x,y)$.

### Triangular QW

Let us describe first the dynamics corresponding to the massless case. Again, we follow the same procedure as in^18^. The triangles have equilateral sides labeled by *k* = 0, 1, 2. The two-dimensional spinors live on the edges shared by adjacent triangles. We denote them by $$\psi (t,v,k)=(\begin{array}{l}{\psi }^{\uparrow }(t,v,k)\\ {\psi }^{\downarrow }(t,v,k)\end{array})$$, with *v* a triangle and *k* a side. Therefore, the position at the lattice will be labeled by (*v*, *k*). The evolution of the Triangular QW is defined as the composition of three operators. The first operator is the application of the 2 × 2 unitary matrix *U*_*i*_(*t*, *v*, *k*), defined in Methods, to each two-dimensional spinor on every edge shared by two neighboring triangles. The second operator, *R*, simply rotates every triangle anti-clockwise. The third operator is just the application of the unitary matrix ${U}_{i}^{\dagger }(t,v,k+1)$ again at each edge shared by two neighboring triangles, where the addition *k* + 1 is understood modulo 2. Altogether, the Triangular QW evolution is given by:

47 $$\begin{array}{rcl}\psi (t+\varepsilon /3,v,k) & = & {U}_{i}^{\dagger }(t,v,k)[{P}^{\uparrow }{U}_{i}(t,v,k-1){e}^{-i\varepsilon {\gamma }_{i}}\psi (t,v,k-1)\\  &  & \oplus \,{P}^{\downarrow }{U}_{i}(t,e(v,k),k-1){e}^{-i\varepsilon {\gamma }_{i}}\psi (t,e(v,k),k-1)]\equiv {W}_{i}(t)\psi (t)\end{array}$$

where *P*^↑^ and *P*^↓^ are the projectors over the upper and lower component of the spinor, respectively, and *e*(*t*, *v*, *k*) is the neighbor of triangle *v* alongside *k* at fixed time *t*. We define one timestep of the evolution by the composition of the three operators *W*_*i*_, and include the mass term, as follows

48 $$\psi (t+\varepsilon )={e}^{-i\tilde{m}\varepsilon {\sigma }^{z}}({W}_{2}{W}_{1}{W}_{0})\psi (t)$$

By expanding this equation up to first order in *ε*, after a tedious but straightforward computation, one arrives to the following equation in the continuum limit:

49 $$\begin{array}{rcl}{\partial }_{t}\psi  & = & ({U}_{0}^{\dagger }{\sigma }^{z}{U}_{0}-\frac{1}{2}{U}_{1}^{\dagger }{\sigma }^{z}{U}_{1}-\frac{1}{2}{U}_{2}^{\dagger }{\sigma }^{z}{U}_{2}){\partial }_{x}\psi +\frac{\sqrt{3}}{2}({U}_{1}^{\dagger }{\sigma }^{z}{U}_{1}-{U}_{2}^{\dagger }{\sigma }^{z}{U}_{2}){\partial }_{y}\psi \\  &  & +\,{\partial }_{x}({U}_{0}^{\dagger }{\sigma }^{z}{U}_{0}-\frac{1}{2}{U}_{1}^{\dagger }{\sigma }^{z}{U}_{1}-\frac{1}{2}{U}_{2}^{\dagger }{\sigma }^{z}{U}_{2})\psi +\frac{\sqrt{3}}{2}{\partial }_{y}({U}_{1}^{\dagger }{\sigma }^{z}{U}_{1}-{U}_{2}^{\dagger }{\sigma }^{z}{U}_{2})\psi -i\tilde{m}{\sigma }^{z}\psi \end{array}$$

where the above terms appear from an expansion at order *O*(*ε*). Notice that, if we define ${B}^{x}\equiv ({\beta }^{0}-\frac{1}{2}{\beta }^{1}-\frac{1}{2}{\beta }^{2})$, and ${B}^{y}\equiv \frac{\sqrt{3}}{2}({\beta }^{1}-{\beta }^{2})$, Eq. (49) adopts the desired form of (17).

#### Numerical simulations

In order to illustrate how the above scheme can be used to describe the dynamics of a particular system, we have computed the behaviour of a massless fermion in a (2 + 1)- dimensional spacetime black hole, whose metric in Lemaître coordinates is given by:

50 $$d{s}^{2}=(1-\frac{{r}_{s}}{r})d{t}^{2}-\frac{d{\rho }^{2}}{1-\frac{{r}_{s}}{r}}-{r}^{2}d{\theta }^{2},$$

where $r={(\frac{3(\rho -t)}{2})}^{\frac{2}{3}}{r}_{s}^{\frac{1}{3}}$, and *r*_*s*_ is the Schwarzschild radius. To simplify the simulations and the plots, we have not considered the angular motion, so that the variation in *θ* is zero. This allows us to describe the QW probability density in the plane (*t*, *x*), where *x* plays the role of *ρ*. The deformation Λ(*t*, *x*) to induce the former metric reads:

51 $${\rm{\Lambda }}(t,x)=(\begin{array}{cc}\frac{\sqrt{r(t,x)}}{{r}_{s}} & 0\\ 0 & \frac{1}{r(t,x)}\end{array}).$$

**Figure 1:**
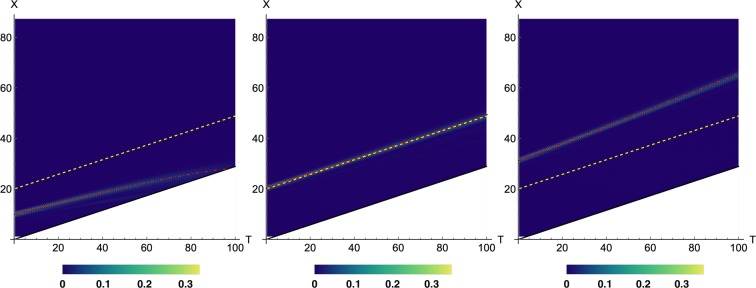
Probability density of a QW in the plane (*t*, *x*), compared with the classical geodesic (dot dashed red line). Dashed yellow line refers to the black hole horizon. Coordinates *T* and *X* are given by $T=\frac{\sqrt{3}}{6}\varepsilon t$, where the factor $\frac{\sqrt{3}}{6}$ is a necessary rescaling of the time coordinate^18^, and *X* = *εx* with $\varepsilon =\frac{1}{3}$. The number of time steps is *t* = 300. The initial condition is *ψ*(0, *v*, 1) = *g*(*v* − *v*_0_)(1, 1)^*T*^ where *g*(*v*) is a Gaussian function with *σ* = 3. See the text for an explanation of the different panels.

In Fig. 1 we can observe the dynamics of the walker in the projected plane (*t*, *x*). Depending on the initial position of the walker, the trajectories in the spacetime vary. The event horizon is given by ${r}_{h}=\frac{\sqrt{3}}{6}\varepsilon t+\frac{2}{3}{r}_{s}$. Therefore, when the particle is initialized inside the horizon with ${x}_{{v}_{0}}=9.94$ (left panel), the QW ends up in the singularity. On the other hand, if the QW starts exacly at the horizon (central panel), the probability distribution will follow the horizon trajectory. Finally, if the initial state lies outside the horizon with ${x}_{{v}_{0}}=31.31$ (right panel), it propagates away from the singularity. These results are in agreement with^30^, in which they study a QW with the same metric in (1 + 1)- dimensional spacetime.

## Discussion

We introduced a Quantum Walk (QW) over the honeycomb and the triangular lattice. In both cases, our starting point was the possibility to rewrite the targeted Hamiltonian as a sum of momentum operators along the three relevant directions of the lattice, each weighted by a suitably chosen gamma matrix. This procedure has been introduced in^18^ — our targeted Hamiltonian was then that of the Dirac equation, which we recovered in the continuum limit. In the present work, we realized that due to the linear dependence of the three preferred directions of the honeycomb and the triangular lattices, one could also obtain the Hamiltonian of the Dirac equation under an arbitrary change of coordinates. We emphasized that applying the same procedure, but for the square lattice, only allows for a very limited set of changes of coordinates.

Then, by making the gamma matrices to be spacetime dependent, we obtained the Curved Dirac equation in an arbitrary background metric. Overall, the QW hereby constructed over the honeycomb and the triangular lattices thus recovers, in the continuum limit, the Dirac equation in curved (2 + 1)– dimensional spacetime. We believe that the duality between changes of metric, and changes of gamma matrices weighting non linearly-independent momentum operators, is profound and may lead to further developments.
